# Main-Chain Benzoxazines Containing an Erythritol Acetal Structure: Thermal and Degradation Properties

**DOI:** 10.3390/molecules28207234

**Published:** 2023-10-23

**Authors:** Huili Yang, Yanqin Du, Guangshe Zhang, Ling Han, Longgui Zhang, Riwei Xu

**Affiliations:** 1 Key Laboratory of Carbon Fiber and Functional Polymers, Beijing University of Chemical Technology, Ministry of Education, Beijing 100029, China; ch_yhl@163.com (H.Y.); d1584946123@163.com (Y.D.); zgshe0622@163.com (G.Z.); 2 SINOPEC (Beijing) Research Institute of Chemical Industry Co., Ltd., Beijing 100013, China; zhanglg.bjhy@sinopec.com

**Keywords:** benzoxazine, erythritol, acetal, degradation, bio-based

## Abstract

In this paper, the bio-based raw material erythritol was used to introduce an acetal structure into the benzoxazine resins. The benzoxazine-based resins containing an erythritol acetal structure could be degraded in an acidic solution and were environmentally friendly thermosetting resins. Compounds and resins were characterized by ^1^H nuclear magnetic resonance (^1^H NMR) and Fourier-transform infrared (FT-IR) analyses, and melting points were studied by a differential scanning calorimeter (DSC); the molecular weight was analyzed by gel permeation chromatography (GPC). The dynamic mechanical properties and thermal stability of polybenzoxazine resins were studied by dynamic mechanical thermal analysis (DMTA) and a thermogravimetric analyzer (TGA), respectively. The thermal aging, wet-heat resistance, and degradation properties of polybenzoxazine resins were tested. The results showed that the polybenzoxazine resins synthesized in this paper had good thermal-oxidative aging, and wet-heat resistance and could be completely degraded in an acidic solution (55 °C DMF: water: 1 mol/L hydrochloric acid solution = 5:2:4 (*v*/*v*/*v*)).

## 1. Introduction

For decades, with the continuous development of science, technology, and industry, the performance requirements of various materials have become higher and higher. Much research has been performed in the area of benzoxazine, a high-performance thermosetting resin. During the curing process, benzoxazine (BOZ) does not emit small molecules and it does not shrink during the polymerization process [1,2,3]. It has a highly crosslinked three-dimensional network, high dimensional stability, good mechanical properties, excellent thermal stability [4,5,6], and low water absorption [7,8]. It is an irreplaceable material in the polymer industry and is widely used in navigation, aviation, aerospace, composite materials, and other fields [9,10,11].

Polybenzoxazine (PBZ) resins are insoluble after cross-linking. At present, the main method to treat thermosetting resin materials is landfill, which pollutes the environment. In addition, the use of incineration, pyrolysis, and other methods consumes a lot of energy and brings secondary pollution. These methods are only simple treatments and do not solve the problem of material recovery at the root. Therefore, it is necessary to improve the recycling of materials through molecular structure design. Recyclable benzoxazine resins are mainly divided into two categories. One is the synthesis of self-repairing resin, and reversible dynamic covalent bonds can be broken and recombined by external excitation. In nature, many organisms have self-healing behaviors, and scientists are also developing self-healing materials. In the absence of external healing agents, polymer materials can self-heal physical damage caused by environmental or mechanical factors through dynamic reversible crosslinking, including covalent reactions, such as the Diels–Alder reaction [12], transesterification reaction [13], and disulfide bonding [14], and noncovalent interactions, such as hydrogen bonding [15]. For example, Rucigaj and his colleagues introduced network flexibility and fluidity into the copolymer system by selecting aliphatic molecules of stearylamine and jeffamine, and designed and crosslinked two benzoxazines based on bisphenol acids. They formed smart polymers with shape memory and self-healing abilities [16]. Polybenzoxazine showed satisfactory recycling, reshaping, and self-healing properties in a study by Salendra and his colleagues because of the strong hydrogen bonding interactions and cleavage reformation of the S–S bond in vitrimers [17]. Eco-friendly sustainable poly (benzoxazine-co-urethane) designed by Sriharshitha and his colleagues had excellent self-healing abilities due to the interaction of supramolecular hydrogen bonds, which could extend shelf life and reliability in the form of self-healing coatings and composite materials [18].

The other way is that degradable structures are introduced into the molecular structure of benzoxazine, which enables the cross-linked polybenzoxazine resins to break at degradable structures under specified chemical environments and permit polybenzoxazine resins available for further recycling after usage. Many scholars have introduced unstable bonds such as acetal bonds, ester bonds, Schiff base bonds, disulfide bonds, hexahydrotriazine structures, borate bonds, and other dynamic chemical bonds into thermosetting resins so that they can be degraded and recycled effectively. These unstable bonds will break under certain conditions, such as heat, light, and specific pHs, which will degrade and recycle thermosetting resin [19,20,21,22,23,24,25,26,27,28]. Liu and his colleagues designed a degradable Schiff base benzoxazine thermosetting material with a high glass transition temperature [29]. Cured benzoxazine can be chemically degraded under acidic conditions, and it undergoes controlled degradation when temperature, acidity, and solvent change. Adjaoud and his colleagues designed a high-glass-transition-temperature (*T*_g_) and degradable isosorbide-based polybenzoxazine vitrimer [30]. The material was highly stable in pH-neutral water, even at 80 °C for 60 days, but due to the structure of isosorbide, obvious degradation was observed under acidic or alkaline conditions.

A remarkable feature of benzoxazine-based polymers is that they have low system shrinkage or even no volume shrinkage during curing. However, several shortcomings are also associated with benzoxazine resins including brittleness and a low molecular weight of prepolymer, which makes it difficult to process into films. Because of the rich design flexibility of the molecular structure, it opens up a broad opportunity for further improving the properties [31]. Researchers have developed a new structure of benzoxazine by using the flexible molecular design of BOZ. That is, the main chain of a synthetic monomer or its copolymer contains benzoxazine rings, which is called main-chain benzoxazine (MCBP). Main-chain benzoxazine prepolymers tend to cross-link to obtain excellent strength and flexibility, and the material after heating and curing is still a thermosetting polymer, which has good application prospects, for example, being used in electronic packaging, printed circuit boards, aviation, and film materials. Liu et al. synthesized a series of bio-based fluorine-free main-chain benzoxazine precursors by a Mannich condensation reaction. The newly developed bio-based main-chain polybenzoxazine resin showed good thermal stability and low surface free energy, which implies great application potential as a high-performance resin matrix [32]. By knowing the molecular weight distribution of the prepared mixture of the polybenzoxazine precursor, its influence on the thermal, mechanical, and viscoelastic properties of the resin during processing and polymerization could be investigated. Ručigaj et al. found that the mixture of precursors of main-chain polybenzoxazine with higher average molecular weight and wider molecular weight distribution showed faster curing and higher crosslinking density of cured resin, thus producing main-chain polybenzoxazine with improved properties [33]. Li et al. synthesized several polybenzoxazines with different molecular structures from cardanol. The mechanical properties test showed that the impact strength of the double-capped cardanol backbone PBZ was 243% higher than that of traditional PBZ [34]. The crosslinking density, thermal stability, carbon residue rate, and molecular diversity were enhanced after the main chain polybenzoxazine was terminated.

Many thermosetting resin raw materials come from non-renewable resources such as bisphenol A, which has adverse effects on the environment and the human endocrine system. Considering the limited reserves of fossil resources and environmental benefits, people have begun to look for bio-based materials with low prices and high environmental benefits, such as lignin, cellulose, vanillin, and other bio-based resources that have been used to prepare thermosetting resins. Erythritol ((R, S)-1,2,3,4-butanetriol) is a filling sweetener in sugar alcohol, which has no carcinogenicity and no blood sugar reaction. Martin et al. found that erythritol had no metabolism and almost no irritation in the digestive system by measuring the labeled erythritol in vitro. Most erythritol is absorbed into the blood through the small intestine and excreted through urine, and a small amount of unabsorbed erythritol, which is not easy to ferment, will enter the colon [35]. Erythritol can also be used as a component and additive in polymer reactions to produce resin, polyether, and polyester. Yuan et al. synthesized a biodegradable epoxy monomer containing acetal structure [36], which was derived from lignin derivative vanillin, bio-based polyol erythritol, and biogenic epichlorohydrin. When the acetal structure is alkaline and neutral, it is stable even in a wet-heat aging test, but it can be completely degradable under acidic conditions, and the degradation rate is accelerated with an increase in the polarity of organic solvents.

**Scheme 1:**
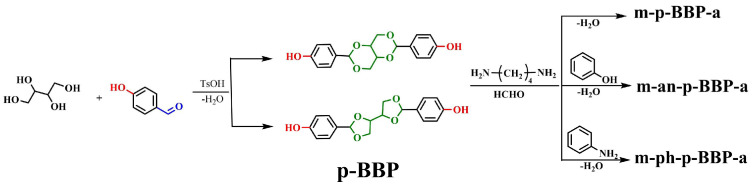
Synthetic route of benzoxazine-based resins containing erythritol acetal structure.

**Figure 1:**
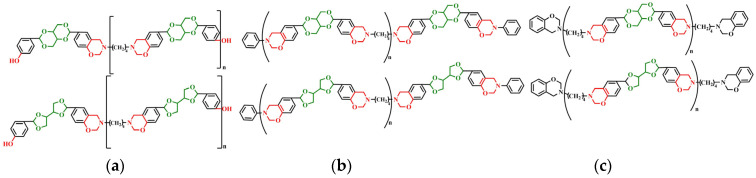
Structure of benzoxazine-based resins: (**a**) m-p-BBP-a; (**b**) m-an-p-BBP-a; (**c**) m-ph-p-BBP-a.

In this work, the acetal compound p-BBP was synthesized from the bio-based raw material erythritol. Erythritol widely exists in nature and is listed in the Generally Recognized as Safe list by the Food and Drug Administration, which is widely used for baked foods. The acetal structure is stable in alkaline and neutral conditions, and it will be hydrolyzed in an acidic solution. Then, with the acetal compound and butanediamine and paraformaldehyde as raw materials, benzoxazine-based resins with different structures of erythritol main chain (m-p-BBP-a, m-an-p-BBP-a, and m-ph-p-BBP-a) (Figure 1) were synthesized by aniline or phenol capping (Scheme 1). The polybenzoxazine resins were studied by several measuring methods. The results showed that polybenzoxazine resins were chemically degradable and could be degraded in an acidic solution, which is beneficial for recycling waste and saving resources, and has good environmental benefits.

## 2. Results and Discussion

### 2.1. Synthesis and Characterization m-p-BBP-a, m-an-p-BBP-a, and m-ph-p-BBP-a

**Figure 2:**
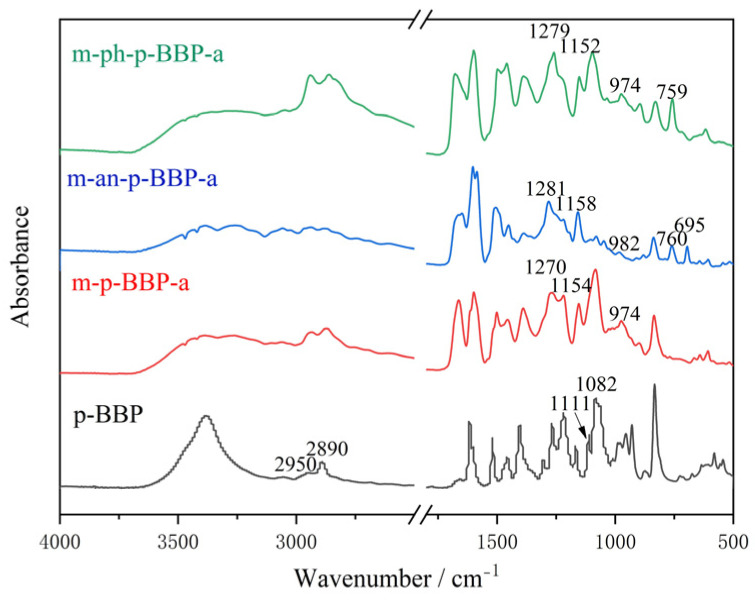
FT-IR spectrum of p-BBP and benzoxazine-based resins.

FT-IR spectrums of p-BBP and benzoxazine-based resins monomers are shown in Figure 2. For m-p-BBP-a, there was a characteristic peak at 3515 cm^−1^ belonging to Ar-O-H, an absorption vibration peak of 1389 cm^−1^ in the product belonging to CH_2_- in diamine, an absorption vibration peak of 1501 cm^−1^ belonging to benzene ring C-C, and absorption vibration peaks of 974 cm^−1^, 1154 cm^−1^, and 1270 cm^−1^ belonging to Ar-O-C, which can explain the existence of oxazine rings. At 1083 cm^−1^, there were characteristic absorption peaks of tertiary carbon C-O of five-membered and six-membered acetal rings connected to two O atoms, and at 2935 cm^−1^ and 2872 cm^−1^, there were -CH- asymmetric and symmetric stretching vibration peaks of five-membered and six-membered acetal rings -CH_2_-. At 3386 cm^−1^, there was an -OH stretching vibration peak, which indicated that both ends of benzoxazine were -OH, indicating that m-p-BBP-a was synthesized. For m-an-p-BBP-a, there was no notable difference in the FT-IR spectroscopy compared with m-p-BBP-a. The characteristic absorption peaks at 760 cm^−1^ and 695 cm^−1^ belonged to monosubstituted benzene rings, which could indicate that aniline had been blocked, indicating that m-an-p-BBP-a had been synthesized. Compared with m-p-BBP-a, the characteristic absorption peak at 759 cm^−1^ belonged to the ortho-substitution of the benzene ring, which could indicate that phenol had been blocked, indicating that m-ph-m-BBP-a had been synthesized.

**Figure 3:**
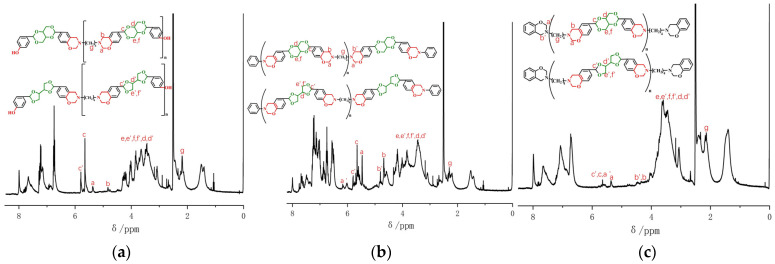
^1^H NMR spectrum of three benzoxazine-based resins: (**a**) m-p-BBP-a; (**b**) m-an-p-BBP-a; (**c**) m-ph-p-BBP-a.

Figure 3 shows the ^1^H NMR spectrum of three types of benzoxazine-based resins. As can be seen from Figure 3a, δ = 5.38 ppm was the characteristic peak of H_a_ on CH_2_ in the middle of the N-CH_2_-O structure, δ = 4.83 ppm was the characteristic peak of H_b_ on CH_2_ in N-CH_2_-Ar, and δ = 6.77 ppm to 7.96 ppm were the characteristic peaks of H on the benzene ring. From the figure, it can be seen that the products had two structures. δ = 5.78 ppm was the characteristic peak of H_c’_ on tertiary carbon C-H in a five-membered acetal ring, δ = 5.65 ppm was the characteristic peak of H_c_ on tertiary carbon C-H in a six-membered acetal ring, and the integral area ratio was 0.40:0.60. δ = 3.05 ppm to 4.26 ppm, assigned to the acetal spiro ring. (δ = 3.30 ppm and δ = 2.50 ppm were H characteristic peaks of water and deuterated dimethylsulfoxide DMSO-*d*_6_, respectively).

Table 1 is a summary of the chemical shifts of characteristic H of benzoxazine-based resins. It can be observed that the chemical shift of terminal group H was greater than the main chain after blocking, and the chemical shift of the oxazine ring of the main chain moved to the low field, which was caused by the electron-withdrawing and unmasking effects of the benzene ring.

**Figure 4:**
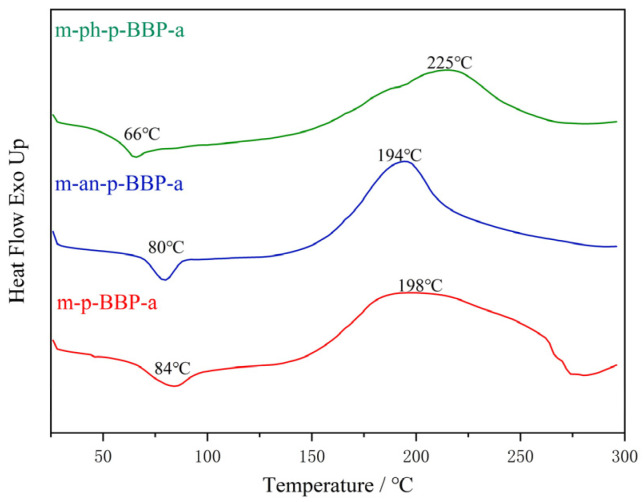
DSC curve of benzoxazine-based resins.

As shown in Figure 4, the DSC curves of benzoxazine-based resins showed that the melting point *T*_m_ of m-p-BBP-a was 84 °C and the peak curing temperature *T*_p_ was 198 °C, *T*_m_ of m-an-p-BBP-a was 80 °C and *T*_p_ was 194 °C, and *T*_m_ of m-ph-p-BBP-a was 66 °C and *T*_p_ was 225 °C. Both molecular structure and molecular weight affected the *T*_m_ of benzoxazine-based resins, and the molecular structure had the greatest influence. The free phenolic hydroxyl groups of m-p-BBP-a formed intermolecular hydrogen bonds, making *T*_m_ higher than the other two benzoxazine-based resins. The electron-withdrawing group of benzoxazine end capped by aniline could reduce the bond energy on the benzoxazine ring and the curing temperature, which was beneficial to the ring-opening polymerization of benzoxazine. Free phenolic hydroxyl groups in m-p-BBP-a could accelerate ring-opening polymerization and reduce curing temperature. There are two reasons why the curing temperature of m-ph-p-BBP-a was high. One is that phenol has excellent reactivity, which can also provide reaction sites when it is cured and crosslinked by heat. The other is that the molecular weight increased and the fluidity decreased after phenol end-capping, which hindered polymer chains from approaching each other and the curing temperature moved to a high temperature. In practical application, rheological properties are also important. The relationship between the viscosity and temperature of benzoxazine-based resins is shown in the Supplementary Document, as shown in Figure S7, which is affected by many factors and will not discussed in the present paper.

**Figure 5:**
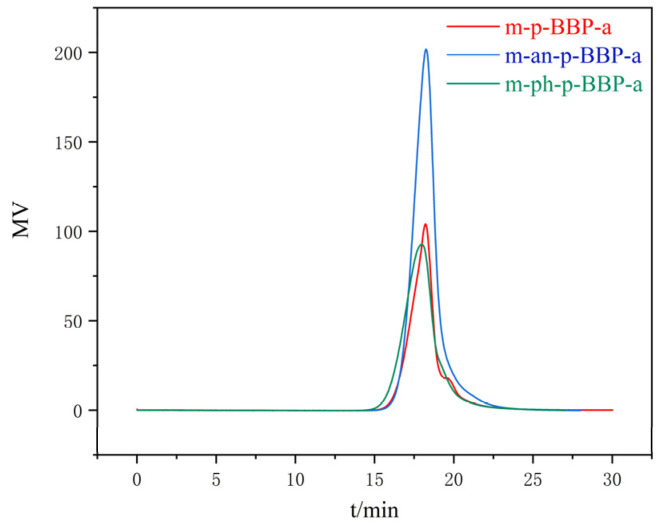
Molecular weight distribution diagram of benzoxazine-based resins.

There are many factors that affected the molecular weight of the main-chain benzoxazine synthesized in this paper, among which the ratio of raw materials, solvent, and temperature were the main factors. The ratio was mainly to control the equimolar ratio of functional group phenol to an amino group to obtain a high molecular weight prepolymer, and the ratio of amine, phenol, and aldehyde was generally 1:1:4 [37]. Phenol or aniline was a monofunctional monomer. As long as it was added, the molecular weight inevitably decreased. Secondly, the solubility of the prepolymer needed to be considered. The interaction between molecules was strong, the molecular weight was large, the solubility was reduced, and it was difficult to continue the reaction, so it was necessary to choose a suitable solvent. Thirdly, the reaction was affected by temperature. When the temperature was low, the reaction was incomplete, but when the temperature was too high, the molecular weight decreased. Figure 5 is the molecular weight distribution diagram of benzoxazine-based resins. Table 2 is a summary of the relative molecular weight of benzoxazine-based resin monomers, and n is the average degree of polymerization estimated according to the relative molecular weight. It can be seen from Figure 5 that the molecular weight distribution of m-an-p-BBP-a was more concentrated, with the most end-capping structures and the smallest PDI. The molecular weight distribution of m-ph-p-BBP-a was wider than other benzoxazine-based resins, with a longer reaction chain and a higher degree of polymerization, which may have been caused by different reaction rates [38].

### 2.2. Curing Behavior of m-p-BBP-a, m-an-p-BBP-a, and m-ph-p-BBP-a

**Figure 6:**
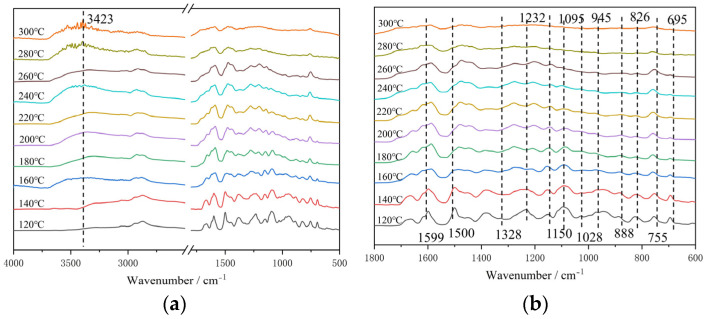
FT-IR spectrum of m-an-p-BBP-a at different temperatures: (**a**) wavenumber range from 4000 to 500 cm^−1^; (**b**) wavenumber range from 1800 to 600 cm^−1^.

**Scheme 2:**
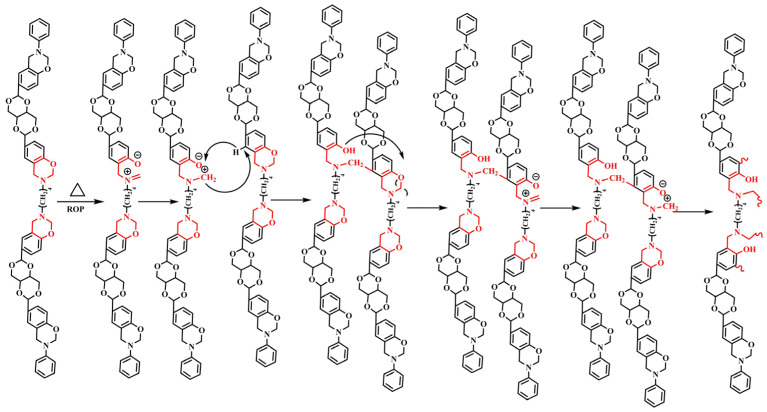
Thermal ring-opening curing mechanism of m-an-p-BBP-a.

Figure 6 shows the infrared curves of m-an-p-BBP-a’s segmented curing. It can be seen from the figure that at the initial stage of curing, the oxazine ring opened, and with the opening of the C-O bond, phenolic hydroxyl groups were formed, and intramolecular or intermolecular hydrogen bonds were formed. The vibration peak of C-N-C in the oxazine ring was at 1150 cm^−1^, and a wide absorption band was generated at 3423 cm^−1^; the characteristic absorption peaks of C-O-C in the acetal ring were at 1095 cm^−1^ and 826 cm^−1^. With an increase in temperature, they gradually weakened. When the temperature rose to 280 °C, the absorption peaks disappeared, and the C-C stretching vibration peak of the benzene ring at 1501 cm^−1^ slowly weakened and gradually moved to a lower wave number direction. The single peak at 1599 cm^−1^ gradually weakened into two broad peaks, and the characteristic absorption peaks of the 1,2,4-trisubstituted benzene ring at 888 cm^−1^ and 826 cm^−1^ also gradually changed. The characteristic peak of the 5-tetra-substituted benzene ring also moved to the lower wave number direction. It can be seen that the newly-appeared phenolic hydroxyl -OH catalyzed the ring opening of the benzoxazine ring, in which the imine ion was electrophilicly replaced by another oxazine molecule, forming the Mannich bridge structure, and benzoxazine was crosslinked into polybenzoxazine, and the curing reaction took place. The characteristic peaks at 1028 cm^−1^ and 1232 cm^−1^ were the stretching vibration peaks of the C-O-C bond. Through continuous heating and curing, the C-O-C bond of the oxazine ring gradually opened, and the peak intensity slowly decreased until it finally disappeared. The characteristic peak at 945 cm^−1^ was the vibration peak of the benzene ring connected with oxazine ring, which slowly decreased with the curing reaction until it finally disappeared completely, indicating that the curing reaction had been completed. The curing mechanism followed the thermal polymerization of benzoxazine, taking the six-membered ring m-an-p-BBP-a as an example, as shown in Scheme 2. Figures S5 and S6 are the segmented curing infrared curves of m-p-BBP-a and m-p-ph-BBP-a, respectively; the curing mechanism is shown in Scheme S1. The curing mechanism proposed in Scheme 2 is a suggested mechanism, which is also applicable to Scheme S1.

The curing kinetics of benzoxazine-based resins were studied through the non-isothermal DSC test performed under different heating rates of 5, 10, 15, and 20 °C·min^−1^. According to the results of the isothermal DSC curves, the exothermic peak temperatures (*T*_p_) at different heating rates were obtained. The apparent activation energy (*E*) of benzoxazine-based resins could be calculated by the Kissinger (Equation (1)) and Ozawa (Equation (2)) methods.

(1) $$\frac{d\left[\ln\left({\beta /T}_{p}\right)\right]}{d\left({1/T}_{p}\right)}=-E/R$$

(2) $$\frac{d\left(\ln\beta \right)}{d\left({1/T}_{p}\right)}=-1.052E/R$$

R is the gas constant of 8.314 (mol · k); E is the apparent activation energy; T_p_ is the curing peak temperature; β is the curing rate.

The polymer curing reaction order (n) and the peak shape index (S) satisfied Equation (3).

(3) $$n=1.26\sqrt{S}$$

S is the absolute value of the tangent slope ratio of the left and right inflection points of the non-isothermal DSC curve.

The pre-exponential factor and the average value of activation energy satisfied Equation (4).

(4) $$A=\frac{\beta \mathrm{Eexp}\left({E/RT}_{p}\right)}{RT_{p}{}^{2}}$$

A is the pre-exponential factor.

**Figure 7:**
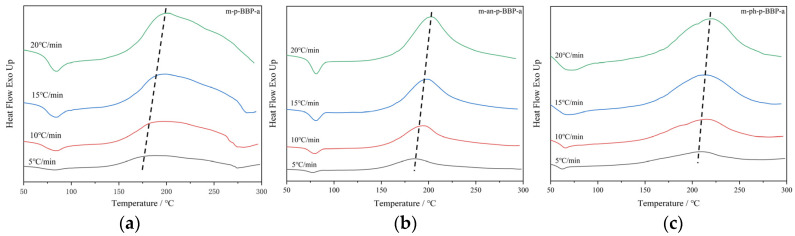
The non-isothermal DSC curves of benzoxazine-based resins: (**a**) m-p-BBP-a; (**b**) m-an-p-BBP-a; (**c**) m-ph-p-BBP-a.

As shown in Figure 7, the curing process of the three benzoxazine-based resins was a melting peak and a curing exothermic peak, and the peak shapes were wide. The curing temperature increased with the increase in the heating rate, the curing exothermic peak moved to the high-temperature direction, and the peak width became wider. This is because the heat energy lagged at a high heating rate and was not transmitted to benzoxazine-based resin molecules in time, and the heat energy released by curing was not detected in time. Moreover, at the same heating interval, the heat preservation time was short, the curing degree was not deep, and the exothermic peak shape was wide. At a low heating rate, the heat energy was transmitted in time, the heat preservation time was long, and the curing degree was deep.

**Figure 8:**
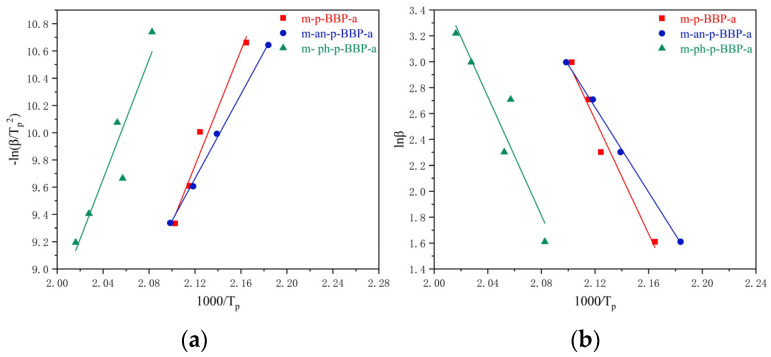
Liner fitting curves of benzoxazine-based resins: (**a**) Kissinger equation; (**b**) Ozawa equation.

Figure 8 shows the linear fitting curve of benzoxazine-based resins. In the case of compound m-ph-p-BBP-a, the linear correlation coefficient was low, and five points were used to draw a linear fitting curve. Table 3 shows the curing kinetic activation energy of benzoxazine-based resins calculated by the linear fitting of the Kissinger and Ozawa equations.

The curing kinetics data of three benzoxazine-based resins are shown in Table 4, which are derived from Tables S1–S4 and Figure S6. The curing kinetics equation of the unblocked main-chain oxazine resin (m-p-BBP-a) was closer to the second-order reaction. After blocking (m-an-p-BBP-a and m-ph-p-BBP-a), the reaction order decreased, which was closer to the first-order reaction.

The activation energies of the three benzoxazine-based resins were all high because the three benzoxazine-based resins were long molecular chains with repeating units. The concentration of oxazine ring was very high in the unit weight of benzoxazine pre-polymer. Moreover, a large number of π-π interactions between benzene rings led to tight packing and restricted the movement of the molecular chains, which may have reduced the molecular mobility and activity during curing [39,40]. When the crosslinking density increased, the molecular mobility further decreased, and more energy was needed to complete the curing. The free phenolic hydroxyl group in m-p-BBP-a was beneficial to further ring-opening polymerization. After the aniline was blocked, the electron-withdrawing group could reduce the bond energy of the oxazine ring, resulting in a decrease in activation energy and curing temperature, which promoted the ring-opening polymerization of the benzoxazine-based resins. When it was cured and crosslinked by heat, the blocking of phenol provided more reaction sites for reactions. At the same time, the molecular weight was increased, the mobility was reduced, and the polymer chains were prevented from approaching each other and so moved the exothermic peak to a higher temperature, and had the highest exothermic enthalpy [41].

### 2.3. Dynamic Mechanical Properties of Polybenzoxazine Resins

**Figure 9:**
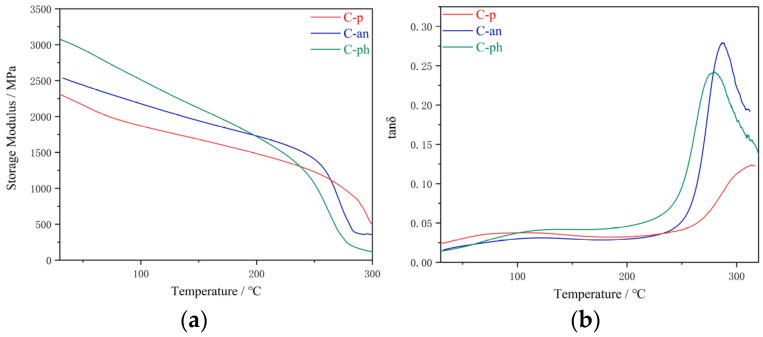
DMTA curves of polybenzoxazine resins: (**a**) storage modulus–temperature curve; (**b**) loss factor–temperature curve.

Figure 9 shows the DMTA test curves of three polybenzoxazine resins: cured m-p-BBP-a (C-p), cured m-an-p-BBP-a (C-an), and cured m-ph-p-BBP-a (C-ph). Figure 9a shows the storage modulus–temperature curve and Figure 9b shows the loss factor–temperature curve. At first, the storage modulus of C-p was 2309 MPa, and it decreased slowly with the increase in temperature. When the temperature rose to 261 °C, the downward trend accelerated, and at 300 °C, the termination modulus was 492 MPa. After aniline blocking, the initial storage modulus was 2534 MPa, and the storage modulus decreased slowly with the increase in temperature. When the temperature rose to 250 °C, the downward trend accelerated, and when it reached 281 °C, it decreased to 445 MPa, and then tended to be flat, and the termination modulus was 357 MPa. After phenol blocking, the initial storage modulus was 3073 MPa, and the storage modulus decreased slowly with the increase in temperature. When the temperature rose to 242 °C, the downward trend accelerated, and when the temperature reached 277 °C, it decreased to 262 MPa, and then the downward speed slowed down, and the termination modulus was 116 MPa. After blocking, the initial storage modulus of the polybenzoxazine resins increased, among which C-ph increased by 764 MPa, indicating that blocking could improve the storage modulus of the polybenzoxazine resins.

The *T*_g_ of the polybenzoxazine resins were obtained from the storage modulus–temperature curve, as shown in Table 5, in which C-p was the highest, which was 313 °C. The results show that the glass transition temperature decreased after blocking.

### 2.4. Thermal Stability of Polybenzoxazine Resins

**Figure 10:**
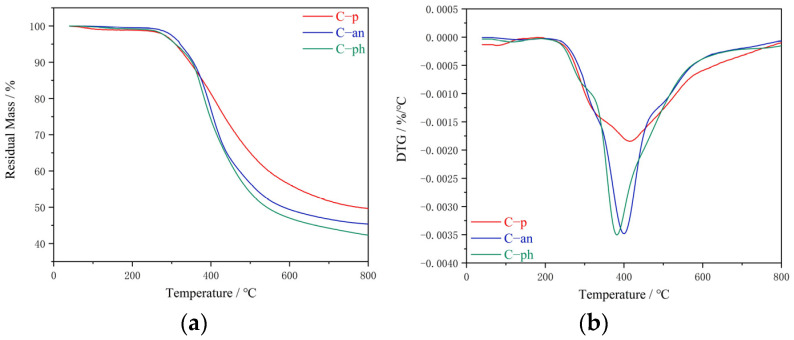
(**a**,**b**) TGA and DTG curves of polybenzoxazine resins (nitrogen).

Figure 10 shows the TGA and derivative thermogravimetry (DTG) curves of polybenzoxazine resins in a nitrogen atmosphere and Table 6 shows the thermogravimetric data of polybenzoxazine resins.

The weight loss at 150 °C may have been caused by the polymer coating on the outer layer and incomplete polymerization of C-p.

*T*_d5%_ is the temperature of 5% weightlessness; *T*_d30%_ is the temperature of 30% weightlessness; *T*_d max_ is the temperature at which the weight loss rate of the sample was the fastest; *Y*_c_ is the carbon residue rate at 800 °C; *T*_s_ is the statistical heat resistance index [36], which could qualitatively compare the thermal stability and satisfy Equation (5):

(5) $$T_{s}=0.49\left(T_{d5}+0.6\left(T_{d30}-T_{d5}\right)\right)$$

According to [42], the flame retardant performance of polybenzoxazine resins can be further evaluated by limiting oxygen index (LOI), and the calculation formula is as shown in Equation (6):

(6) $$\mathrm{LOI}=17.5+0.4Y_{c}$$

*Y*_c_: carbon residue rate at 800 °C. 

It can be seen from Table 6 that the *T*_d5%_ and *T*_d30%_ of polybenzoxazine resins were both very high in a nitrogen atmosphere. The structural rigidity of spiroacetal improved the thermal stability, but the *Y*_c_ and *T*_s_ of C-an and C-ph were smaller than C-p, indicating that the thermal stability decreased after the end-capping. This may be due to the end group structure in the end-capping system in the mixture being unstable and easily decomposed by heat.

The LOI values of polybenzoxazine resins are shown in Table 7, and C-p had the largest LOI value and the best flame retardancy.

The introduction of a spirocyclic skeleton into the main-chain structure of polybenzoxazine resins could significantly improve its thermal stability, so the initial thermal decomposition temperature and carbon residue rate of the three were very high. The large-volume spiro ring structure was rigid. This structure had multiple bonds, which could reduce main-chain breakage at high temperatures. Together with numerous intramolecular and intermolecular H bonds in C-p, they formed a highly crosslinked network structure of polybenzoxazine, so its thermal stability was higher than the other two polybenzoxazine resins. The end group structure in the end-capping system was unstable and easily decomposed by heat, and the heat resistance was slightly reduced.

**Figure 11:**
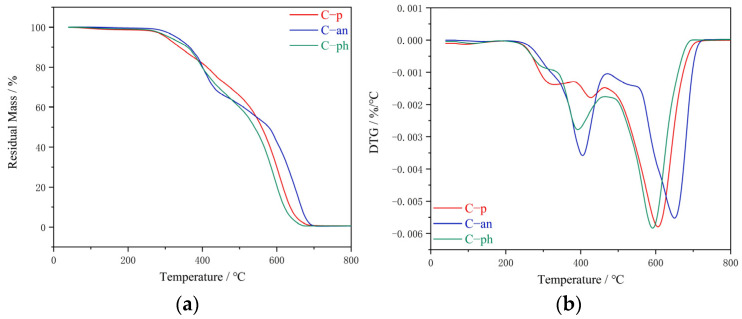
(**a**,**b**) TGA and DTG curves of polybenzoxazine resins (air).

Figure 11 shows the TGA and DTG curves of polybenzoxazine resins in an air atmosphere and Table 8 shows the thermogravimetric data of polybenzoxazine resins.

It can be seen from Table 8 that the Ts of C-an and C-ph were smaller than that for C-p in an air atmosphere. However, the *Y*_c600_ of C-an was higher than that for C-p and C-ph, which showed that the stability of aniline was improved in an air atmosphere within a certain temperature range. The *Y*_c600_ of C-an was higher than for that without end-capping. The reason for this is that the stability of the oxazine main-chain structure and the polymerization degree of oxazine after end-capping were low, so it had higher cross-linking density and network regularity, and a high carbon residue rate at high temperatures.

### 2.5. Thermal Oxidation Aging of Polybenzoxazine Resins

Table 9 shows the data on the thermal oxidation aging of polybenzoxazine resins. It can be seen from the table that end-capping improved the thermal oxidation aging resistance. At 250 °C, the weight loss of three kinds of polybenzoxazine resins was very small, but the *T*_g_ also affected the use temperature of resins. The *T*_g_ of C-p was the highest, which was 313 °C, and it could be used for a long time at 250 °C. After blocking, the *T*_g_ of C-an was 284 °C, the *T*_g_ of C-p was 276 °C, and the blocked polybenzoxazine resins could be used for a long time at 200 °C. However, the resin had a large weight loss rate after raising the temperature, which is not suitable for long-term use.

### 2.6. Wet-Heat Resistance of Polybenzoxazine Resins

**Figure 12:**
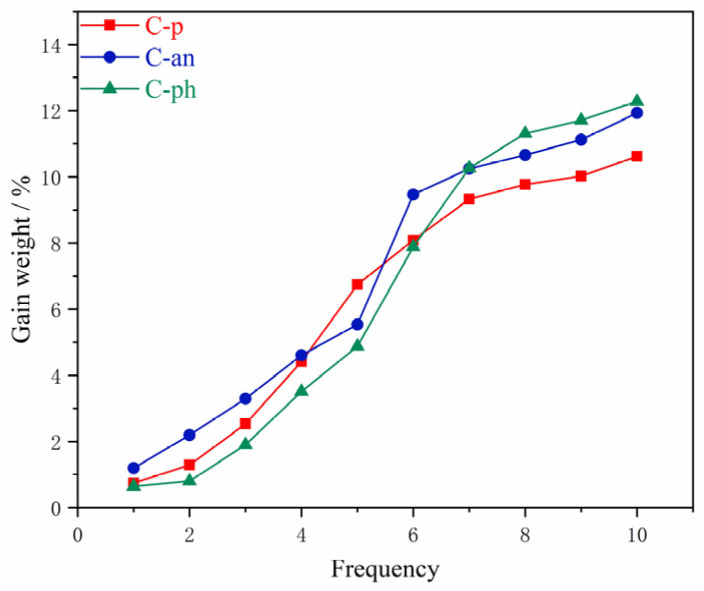
Humidity and heat resistance test curves of polybenzoxazine resins.

The water absorption of resin under wet conditions affects the structural size and physical properties of materials. Figure 12 is the curve of the humidity and heat resistance of polybenzoxazine resins. It can be seen that the weight of the resin increased after being soaked in ultra-pure water at 100 °C, but all by less than 13%, and they finally tended to be flat and within the allowable range. The water absorption was lower in a short time after phenol end-capping because the phenolic hydroxyl group in the molecule promoted the curing, made the cross-linking closer, and effectively inhibited the diffusion of water molecules in the resin matrix.

### 2.7. Degradation Properties of Polybenzoxazine Resins

Acetal is a diether, which can exist stably in alkaline, oxidant, and reducing agents, but it hydrolyzes in acidic solutions to generate original aldehydes and alcohols. The polybenzoxazine resins could also be degraded under acidic conditions. The cyclic acetal structure needed to open two bonds to degrade, and the reclosing of acetal formed a competitive reaction with the breaking of the second bond of acetal, which was not conducive to the breaking of the polymer network, so the process was slow and some oligomer fragments were produced. The degradation properties of cured resins were studied by placing the cured product in an environment with solvent, acid type and concentration, and temperature as variables.

#### 2.7.1. Different Acid Concentrations

**Figure 13:**
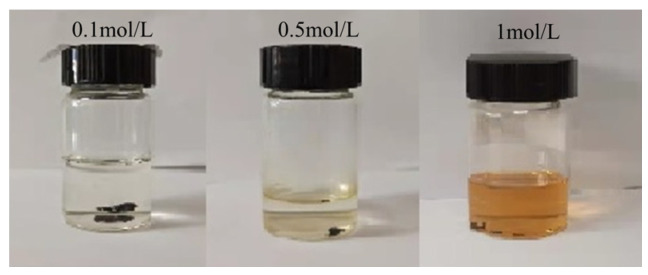
Degradation of C-p in different concentrations of acid.

Figure 13 shows the degradation of C-p in different hydrochloric acid concentrations. 

Table 10 shows the degradation data of C-p in different acid concentrations. The degradation rate was 1 mol/L > 0.5 mol/L > 0.1 mol/L from big to small, and it could be concluded that the higher the acid concentration, the faster the degradation rate.

#### 2.7.2. Different Acid Types

**Figure 14:**
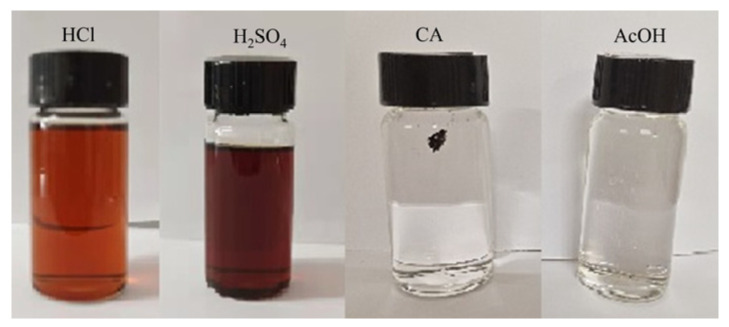
Degradation of C-p in different kinds of acid.

Figure 14 shows the degradation of C-p in different acids.

The degradation data of C-p in different acids are shown in Table 11. At 96 h, the samples in the H_2_SO_4_ solution were completely degraded, the samples in the HCl solution were completely degraded, and the samples in citric acid (CA) and acetic acid (AcOH) were not completely degraded. The degradation rate of C-p in different acids was H_2_SO_4_ > HCl > AcOH > CA. It could be concluded that the stronger the acidity, the greater the degradation rate. However, H_2_SO_4_ was too acidic and corrosive to equipment. Considering safety, hydrochloric acid was the best acid.

#### 2.7.3. Different Organic Solvents

**Figure 15:**
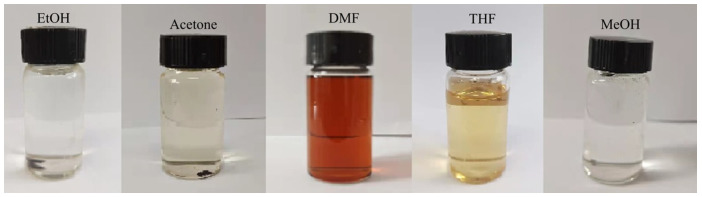
Degradation of C-p in different kinds of solvents.

Figure 15 shows the degradation of C-p in different organic solvents.

Table 12 shows the degradation data of C-p in different organic solvents. The polarity of the solvent was acetone (CP) < tetrahydrofuran (THF) < ethanol (EtOH) < methanol (MeOH) < N,N-dimethylformamide (DMF). The cured product could be completely degraded in DMF, and the degradation rate was the fastest. This was because C-p had the best solubility in DMF. The swelling degree of polybenzoxazine resins and the solubility of polybenzoxazine resins prepolymer were different in different solvents.

#### 2.7.4. Different Polybenzoxazine Resins

Table 13 shows the degradation of different polybenzoxazine resins in a mixed solution.

As can be seen from Table 13, the degradation rate accelerated with an increase in temperature. At the initial stage of degradation, the degradation rate increased with time. Then, it dropped slightly as time went on. After capping, the degradation performance was reduced because the active groups at both ends were reduced, the binding sites with H^+^ were reduced, and the degradation performance and rate were reduced.

## 3. Materials and Methods

### 3.1. Materials

All the reagents were of analytical grade and used without further purification. Erythritol (98%), phenol, sodium bicarbonate (NaHCO_3_), and *p*-toluenesulfonic acid (TSOH) were obtained from Shanghai Maclean Biochemical Technology Co., Ltd. (Shanghai, China); *p*-hydroxybenzaldehyde (98%) was purchased from Shanghai yuanye Bio-Technology Co., Ltd. (Shanghai, China); N,N-Dimethylformamide (DMF) was purchased from Beijing Chemical Factory (Beijing, China). Aniline and paraformaldehyde were purchased from Fuchen Chemical Reagent Co., Ltd. (Tianjin, China); 1,4-butanediamine (99%) was purchased from Acros Organics Co., Ltd. (Geel, Belgium). In addition, p-BBP was synthesized according to the route in Scheme 1 [35]. FT-IR spectrum (KBr) (Figure S1), cm^−1^: 3381 (-phenolic hydroxyl -OH), 2950, 2890 (vibration peak -CH_2_), 1111 (absorption peaks tertiary carbon C-O), 1082 (stretching vibration peak C-O-C). ^1^H NMR spectrum (Figure S2), ppm: 9.49 (phenolic hydroxyl), 7.27–6.76 (benzene ring), 5.80 (tertiary carbon C-H in five-membered acetal ring), 5.64 (tertiary carbon C-H in six-membered acetal ring), 4.31, 4.21, 4.06, and 4.02 (O-CH_2_-C in two kinds of acetal rings), 3.86 and 3.83 (O-CH-C in two kinds of acetal rings). ^13^C NMR spectrum (Figure S3), ppm: 115.36–158.68 (benzene ring), 105.05 and 103.93 (tertiary carbon C-H), 76.52 and 74.39 (acetal rings O-CH), 68.81 and 67.62 (acetal rings O-CH_2_). In Figure S4, the mass spectrum of p-BBP shows that the theoretical molecular weight was 330 g mol^−1^ and the measured [M]^+^ was 330 g·mol^−1^.

### 3.2. Synthesis of m-p-BBP-a

A total of 1.65 g (0.005 mol) of p-BBP, 0.44 g (0.005 mol) of butanediamine, 0.60 g (0.02 mol) of paraformaldehyde, and 20 mL of N,N-dimethylformamide (DMF) was added to a 50 mL three-necked flask, the reaction temperature was set at 115 °C, and it was reacted for 24 h. After the reaction, the solvent DMF was removed from the obtained product by a rotary evaporator and then the reaction product was washed with 0.5 M sodium bicarbonate solution, then filtered, washed with deionized water until neutral, filtered, and dried at 50 °C to obtain the final product, namely, m-p-BBP-a, which was a light yellow powder (yield: 55.8%). FT-IR spectrum (KBr), cm^−1^: 1389 (-CH_2_- absorption vibration peak), 1501 (benzene ring C-C), 974, 1154, and 1270 (oxazine rings Ar-O-C), 1083 (tertiary carbon C-O), 3386 (–OH stretching vibration peak). ^1^H NMR spectrum, ppm: 5.38 (CH_2_ in N-CH_2_-O structure), 4.83 (CH_2_ in N-CH_2_-Ar), 6.77–7.96 (benzene ring), 3.05–4.26 (acetal spiro ring).

### 3.3. Synthesis of m-an-p-BBP-a 

A total of 6.60 g (0.02 mol) of p-BBP, 0.88 g (0.01 mol) of butanediamine, 1.86 g (0.02 mol) of aniline, 2.40 g (0.08 mol) of paraformaldehyde, and 50 mL DMF was added to a 250 mL three-necked flask, and the reaction steps were the same as those for m-p-BBP-a. The prepared benzoxazine was the erythritol bis-p-hydroxybenzaldehyde-butanediamine benzoxazine-based resin terminated by aniline, which is called m-an-p-BBP-a. It is a burnt yellow powder (yield: 61.7%). FT-IR spectrum (KBr), cm^−1^: 1387 (-CH_2_- absorption vibration peak), 1505 (benzene ring C-C), 982, 1158, and 1281 (oxazine rings Ar-O-C), 760 and 695 (monosubstituted benzene rings). ^1^H NMR spectrum, ppm: 5.47 (CH_2_ in N-CH_2_-O), 4.80 (CH_2_ in N-CH_2_-Ar), 6.55–7.70 (benzene ring), 3.18–4.30 (acetal spiro ring).

### 3.4. Synthesis of m-ph-p-BBP-a 

A total of 3.30 g (0.01 mol) of p-BBP, 0.88 g (0.01 mol) of butanediamine, 0.94 g (0.01 mol) of phenol, 1.20 g (0.04 mol) of paraformaldehyde, and 20 mL DMF was added to a 50 mL three-necked flask. The reaction steps were the same as those for m-p-BBP-a. M-ph-p-BBP-a, the erythritol bis-p-hydroxybenzaldehyde-butanediamine benzoxazine-based resin terminated by phenol, was obtained as a yellow powder (yield: 68.4%). FT-IR spectrum (KBr), cm^−1^: 1388 (-CH_2_- absorption vibration peak), 1498 (benzene ring C-C), 974, 1153, and 1260 (oxazine rings Ar-O-C), 3292 (–OH stretching vibration peak). ^1^H NMR spectrum, ppm: 5.43 (CH_2_ in N-CH_2_-O), 4.34 (CH_2_ in N-CH_2_-Ar), 6.70–7.99 (benzene ring), 3.24–4.02 (acetal spiro ring).

### 3.5. Preparation of Polybenzoxazine Resins (m-p-BBP-a, m-an-p-BBP-a, and m-ph-p-BBP-a)

The m-p-BBP-a, m-an-p-BBP-a, and m-ph-p-BBP-a were ground into powder, which was dissolved in tetrahydrofuran with 4 wt% of 4,4′-Bis(3-aminophenoxy)diphenyl sulfone, respectively. Ultrasonic in a numerical control ultrasonic cleaner, evenly mixing, drying, and grinding for later use. The curing was accomplished by undergoing a cure cycle of 110 °C for 2 h, 130 °C for 2 h, 150 °C for 2 h, 170 °C for 2 h, 190 °C for 2 h, 200 °C for 2 h, and 220 °C for 4 h.

### 3.6. Preparation of Thermal Oxidation Aging Samples

The polybenzoxazine resins were polished into standard samples (50 mm × 4 mm × 3 mm) and kept at 250 °C, 300 °C, 325 °C, 350 °C, and 375 °C for 8 h, respectively, weighed and this was repeated 3 times, and the results were averaged.

### 3.7. Preparation of Wet-Heat Resistance Samples

The polybenzoxazine resins were polished into standard samples (50 mm × 4 mm × 3 mm), kept in ultrapure water at 100 °C for 120 h, weighed every 12 h, and this was repeated 3 times, and the results were averaged.

### 3.8. Degradation Properties of Polybenzoxazine Resins 

The polybenzoxazine resins were cut into small pieces of the same size, and each small piece was similar in mass. They were then placed in environments with different acid concentrations, acid types, organic solvents, temperatures, and degradation times, and then cooled, filtered, dried, and weighed. They were tested 3 times and the average value was taken.

The calculation formula of degradation degree C is shown in Equation (7):

(7) $$C=\frac{W_{1}-W_{2}}{W_{1}}\times 100\%$$

C is the degree of degradation (%); W_1_ is the initial cured product mass (mg); W_2_ is the mass of insoluble residue after degradation (mg).

The calculation formula of the degradation rate average value is shown in Equation (8):

(8) $$v=\frac{W_{1}-W_{2}}{t\cdot V}$$

v is the degradation rate average value (mg·h^−1^·mL^−1^); t is degradation time (h); V is the volume of the solution (mL).

#### 3.8.1. Different Acid Concentrations

The mass of each piece was about 20 mg; DMF: water: hydrochloric acid = 5:2:4 (*v*/*v*/*v*). The concentrations of hydrochloric acid were 0.1 mol/L, 0.5 mol/L, and 1 mol/L and the acid concentrations of the mixed solutions were 0.036 mol/L, 0.182 mol/L, and 0.364 mol/L. They were degraded at 85 °C for 6 h. Because DMF has good solubility in a benzoxazine monomer, DMF was used as a solvent to prepare mixed solutions by changing the acid solution. 

#### 3.8.2. Different Acid Types

The mass of each piece was about 5 mg; DMF: water: 1 mol/L different acid solution = 5:2:4 (*v*/*v*/*v*), and the acids were HCl, H_2_SO_4_, anhydrous citric acid CA, and AcOH acetate. The acid concentrations of the mixed solutions were 0.364 mol/L, 0.727 mol/L, 1.091 mol/L, and 0.364 mol/L, which were degraded for 96 h at 55 °C.

#### 3.8.3. Different Organic Solvents

The mass of each piece was about 10 mg. The organic solvent: water: 1 mol/L hydrochloric acid = 5:2:4 (*v*/*v*/*v*). The organic solvents were ethanol (EtOH), acetone (CP), N,N-dimethylformamide (DMF), tetrahydrofuran (THF), and methanol (MeOH), and the acid concentration of the mixed solutions was 0.364 mol/L. The solutions were degraded for 120 h at 55 °C.

#### 3.8.4. Different Polybenzoxazine Resins

The mass of each piece was about 10 mg; DMF: water: 1mol/L hydrochloric acid = 5:2:4 (*v*/*v*/*v*). The acid concentrations of the mixed solutions were 0.364 mol/L.

### 3.9. Characterization 

Nuclear magnetic resonance (NMR) was performed on a Bruker ADVANCE 400 MHz nuclear magnetic resonance spectrometer using DMSO-*d*_6_ as a solvent. 

Fourier-transform infrared (FT-IR) spectra were recorded with Nicolet-IS5 using KBr pellets. 

Elemental analyses were performed on a Vario EL cube. 

Matrix-assisted laser desorption/ionization-time-of-flight (MALDI-TOF) mass spectrometry was conducted on a Bruker Autoflex III time-of-flight mass spectrometer; matrix: α-cyano-4-hydroxycinnamic acid (CCA), ionizing reagent: NaCl, KCl. 

Gel permeation chromatography (GPC) was performed on PL-GPC-220; column temperature: 30 °C, eluant: DMF. 

Differential scanning calorimetric (DSC) analysis was performed on Q100 at a heating rate of 10 °C/min under a nitrogen flow rate of 50 mL/min.

Dynamic mechanical thermal analysis (DMTA) was performed on a Q800 dynamic mechanical analyzer. The measurements were taken under nitrogen in the temperature range of approximately 40–400 °C. A temperature ramp of 5 °C/min was used to determine the storage modulus and damping factor (tan δ) of the material at a frequency of 1 Hz. 

Thermogravimetric analysis (TGA) was performed on TGA Q500 at a heating rate of 10 °C/min under a nitrogen or air purge of 100 mL/min.

## 4. Conclusions

P-BBP was synthesized from biological erythritol and p-hydroxybenzaldehyde. Three kinds of benzoxazine-based resins (m-p-BBP-a, m-an-p-BBP-a, and m-ph-p-BBP-a) containing an erythritol acetal structure were synthesized by the reaction of this raw material with butanediamine and blocking by aniline and phenol. End-capping could improve the storage modulus of the polybenzoxazine resins. The three kinds of polybenzoxazine resins had good thermal stability and great wet and heat resistance in boiling water. C-p could be used for a long time at 250 °C, and the blocked polybenzoxazine resins could be used for a long time at 200 °C. The degradation performance test indicated that the higher the acid concentration, the stronger the acidity, the better the solubility of the prepolymer in solvent, and the faster the degradation rate. After capping, the degradation performance and rate decreased.

## Figures and Tables

**Table 1:** Chemical shift of benzoxazine-based resins.

BZ	-O-CH_2_-N(ppm)		Ar-CH_2_-N (ppm)	
	Chain	End	Chain	End
m-p-BBP-a	5.38		4.83	
m-an-p-BBP-a	5.47	6.17	4.68	4.80
m-ph-p-BBP-a	5.43	5.65	4.34	4.47

**Table 2:** Relative molecular weight of benzoxazine-based resins.

BZ	Mn	Mw	Mz	PDI	n
m-p-BBP-a	2420	4250	7720	1.76	5.63
m-an-p-BBP-a	2210	3570	6190	1.62	3.60
m-ph-p-BBP-a	2990	5910	11290	1.98	8.36

The relative molecular weight is represented for the soluble fraction of three benzoxazine-based resins in this table, especially for m-p-BBP-a.

**Table 3:** Apparent activation energy.

BZ	Kissinger Activation Energy E/kJ/mol	Linear Correlation Coefficient	Ozawa Activation Energy E/kJ/mol	Linear Correlation Coefficient	Average Activation Energy E/kJ/mol
m-p-BBP-a	175.80	0.98401	174.51	0.98528	175.16
m-an-p-BBP-a	128.75	0.99883	129.76	0.99896	129.26
m-ph-p-BBP-a	182.73	0.93722	181.41	0.94203	182.07

**Table 4:** Curing reaction rate equation of benzoxazine-based resins.

BZ	Rate Formula
m-p-BBP-a	dα/dt = 3.05 × 10^19^ exp (−1.75 × 10^5^/RT)(1 − α)^1.66^
m-an-p-BBP-a	dα/dt = 2.04 × 10^14^ exp (−1.29 × 10^5^/RT)(1 − α)^1.29^
m-ph-p-BBP-a	dα/dt = 3.59 × 10^19^ exp (−0.77 × 10^5^/RT)(1 − α)^1.10^

**Table 5:** *T*_g_ of polybenzoxazine resins.

BZ	C-p	C-an	C-ph
*T*_g_/°C	313	284	276

**Table 6:** TGA and DTG data of polybenzoxazine resins (nitrogen).

BZ	*T*_d5%_/°C	*T*_d30%_/°C	*T*_d max_/°C	*Y*_c800_/%	*T*_s_
C-p	309	465	414	49.7	197.3
C-an	322	421	400	45.4	186.9
C-ph	310	416	382	42.3	183.1

**Table 7:** The LOI value of polybenzoxazine resins.

BZ	*Y*_c_/%	LOI
C-p	49.7	37.38
C-an	45.4	35.66
C-ph	42.3	34.42

**Table 8:** TGA and DTG data of polybenzoxazine resins (air).

BZ	*T*_d5%_/°C	*T*_d30%_/°C	*T*_d max_/°C	*Y*_c600_/%	*T*_s_
C-p	302	474	606	30.0	198.5
C-an	330	431	650	42.6	191.4
C-ph	311	443	592	20.7	191.2

*Y*_c600_: carbon residue rate at 600 °C.

**Table 9:** Thermal oxidation aging test data of polybenzoxazine resins (air).

BZ	250 °C/wt%	300 °C/wt%	325 °C/wt%	350 °C/wt%	375 °C/wt%	Total Weightlessness/wt%
C-p	0.56	4.18	6.88	9.39	15.06	36.06
C-an	1.39	3.47	7.87	9.10	12.81	34.65
C-ph	0.48	1.91	3.18	6.28	8.59	20.43

**Table 10:** Degradation data of C-p in different concentrations of acid.

Concentrations	0.1 mol/L	0.5 mol/L	1 mol/L
85 °C 6 h C/%	11	18	72
average value/mg·h^−1^·mL^−1^	0.03	0.05	0.22

**Table 11:** Degradation data of C-p in different kinds of acid.

Acid	HCl	H_2_SO_4_	CA	AcOH
55 °C 96 h C/%	100	100	12	15
average value/mg·h^−1^·mL^−1^	0.24	0.25	0.03	0.04

**Table 12:** Degradation data of C-p in different kinds of solvents.

Solvents	EtOH	Acetone	DMF	THF	MeOH
55 °C 120 h C/%	18	26	100	95	10
average value/mg·h^−1^·mL^−1^	0.03	0.05	0.19	0.18	0.02

**Table 13:** Degradation data of different kinds of polybenzoxazine resins.

BZ	T/°C	t/h	C/%	Average Value/mg·h^−1^·mL^−1^
C-p	25	36	7	0.09
C-p	55	36	12	0.15
C-p	75	36	18	0.23
C-p	75	24	13	0.25
C-p	75	12	9	0.34
C-p	75	8	7	0.40
C-p	75	6	5	0.38
C-an	75	36	16	0.20
C-ph	75	36	9	0.11

## Data Availability

Data presented in this study are available upon request from the corresponding author.

## Supplementary Materials

The following supporting information can be downloaded at https://www.mdpi.com/article/10.3390/molecules28207234/s1: Figure S1. ^1^H NMR of p-BBP. Figure S2. ^13^C NMR of p-BBP. Figure S3. Mass of p-BBP. Figure S4. FT-IR of m-p-BBP-a at different temperatures. Figure S5. FT-IR of m-ph-p-BBP-a at different temperatures. Scheme S1. Thermal ring-opening curing mechanism of m-ph-p-BBP-a. Table S1. The non-isothermal curing DSC data of benzoxazine-based resins. Figure S6. Liner fit of benzoxazine-based resins’ curing temperature. Table S2. The curing temperature of benzoxazine-based resins. Table S3. The non-isothermal DSC curing parameters of benzoxazine-based resins. Table S4. Result computation. Figure S7. Relationship between viscosity and temperature of benzoxazine-based resins (m-p-BBP-a, m-an-p-BBP-a, and m-ph-p-BBP-a).

## Sample Availability

Samples of the compounds are available from the authors.
