# Supplementary material for: Main-Chain Benzoxazines Containing an Erythritol Acetal Structure: Thermal and Degradation Properties
Source: Molecules. 2023 Oct 23;28(20):7234. doi: 10.3390/molecules28207234 (PMC10608865; doi:10.3390/molecules28207234)
Supplement: Supplementary file 1 [file molecules-28-07234-s001.zip › molecules-2608283-supplementary.pdf]

Supporting Information

Snythesis of erythritol bis-p-hydroxybenzaldehyde (p-BBP)

Erythritol bis-p-hydroxybenzaldehyde is synthesized by condensation of aldehyde of p-hydroxybenzaldehyde and hydroxyl group of erythritol [1]. p-BBP was synthesized according to the route in Scheme 1. In a typical experiment, add 12.21 g (0.1 mol) of erythritol and 24.42g(0.2 mol) of p-hydroxybenzaldehyde and 3 wt% p-toluenesulfonic acid into the flask equipped with a Dean-Stark trap with a condenser, thermometer and a stirrer, mix with a certain solvent DMF(N,N-dimethylformamide) and cyclohexane as a water-carrying agent, heat to 115°C at room temperature and reflux until the water yield in the water separator does not increase, and end the reaction. The solvent was removed by rotary evaporation, and after cooling to room temperature, the 5% aqueous solution of $NaHCO_3$ was stirred and filtered, and the precipitate was washed with deionized water until it was neutral. After filtration, it was dried in a vacuum drying oven at 55°C, and p-BBP was obtained as a light yellow powder product. The average yield of five experimental data was 69.2%.

Methods

Nuclear magnetic resonance (NMR) was performed on a Bruker ADVANCE 400 MHz nuclear magnetic resonance spectrometer using DMSO-d_6 as solvent.

Fourier-transform infrared (FT-IR) spectrum was recorded with Nicolet-IS5 using KBr pellets.

Elemental analyses were performed on a Vario EL cube.

Matrix-assisted laser desorption/ ionization time of flight (MALDI-TOF) mass spectrometry was done on a Bruker Autoflex III time-of-flight mass spectrometer; matrix: α-cyano-4-hydroxycinnamic acid (CCA), ionizing reagent: NaCl, KCl.

Differential scanning calorimetric (DSC) analysis was performed on Q100 at a heating rate of 10°C/min under a nitrogen flow rate of 50 mL/min.

Rheological analysis was performed at 100-200°C using DHR-2 at 5°C/min.

Characterization of erythritol bis-p-hydroxybenzaldehyde (p-BBP)

The ^{1}H NMR shows the H of two acetal rings, and the ^{13}C NMR shows that the product has two structures. The p-BBP was characterized by ^{1}H NMR (Figure S1),^{13}C NMR (Figure S2) and FTIR spectroscopy (Figure 2).

Figure 2 is the FT-IR spectrum of p-BBP. From the Figure 2, it can be seen that there is a characteristic peak at 3381 cm^{-1} belonging to phenolic hydroxyl -OH, 2950 cm^{-1} and 2890 cm^{-1} belonging to the asymmetric stretching vibration peak and symmetrical vibration peak of -CH_2 in five-membered and six-membered acetal rings, and 1111 cm^{-1} belonging to the characteristic absorption peaks of tertiary carbon C-O of five-membered and six-membered acetal rings connected to two O atoms. The stretching vibration peak of C-O-C in five-membered and six-membered acetal rings is at 1082 cm^{-1}, and the stretching vibration peak of C=O in p-hydroxybenzaldehyde in raw material disappears at 1672 cm^{-1}. In summary, the obtained spectrums proved the successful synthesis of p-BBP.

The Figure S1 is the ^{1}H NMR spectrum of p-BBP. It can be seen from the figure that $\delta = 9.49$ ppm is the characteristic peak of H in phenolic hydroxyl H_a, $\delta = 7.27$ ppm-6.76 ppm is the characteristic peak of H in benzene ring, $\delta= 5.80$ ppm is the characteristic peak of H in $H_{b'}$ on tertiary carbon C-H in five-membered acetal ring, and $\delta = 5.64$ ppm is the characteristic peak of H on H_b on tertiary carbon C-H in six-membered acetal ring. $\delta= 4.31$ppm, $\delta= 4.21$ppm, $\delta= 4.06$ppm and $\delta = 4.02$ppm are the characteristic peaks of H_d, $H_{d'}$, H_c and $H_{c'}$ on two O-CH_2-C in two kinds of acetal rings, and $\delta = 3.86$ppm and $\delta = 3.83$ppm are the characteristic peaks of H_e and $H_{e'}$, O-CH-C in five-membered and six-membered acetal rings.

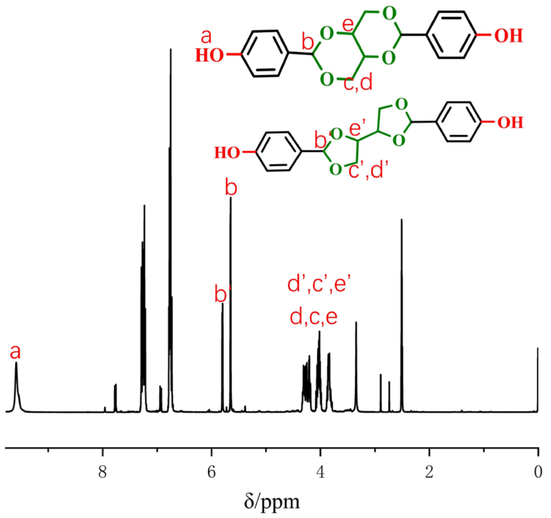

Figure S1 ^{1}H NMR spectrum of p-BBP.

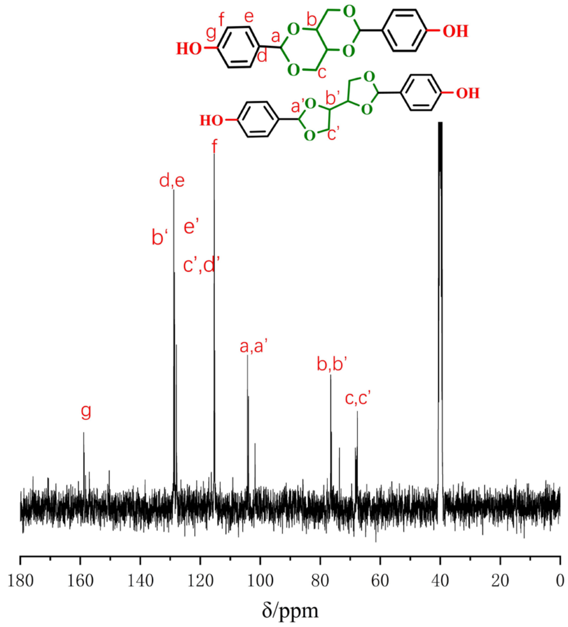

Figure S2. ^{13}C NMR 1H NMR spectrum of p-BBP.

As can be seen from the Figure S2, δ=115.36 ppm-158.68 ppm is the characteristic peak of C_d, C_e, C_f and C_g on benzene ring, δ=105.05 ppm and δ=103.93 ppm is the characteristic peak of C_a and $C_{a'}$ on tertiary carbon C-H in five-membered and six-membered acetal rings. δ=76.52 ppm and δ=74.39 ppm are the characteristic peaks of C_b and $C_{b'}$ on O-CH in five-membered and six-membered acetal rings, and δ=68.81 ppm and δ=67.62 ppm are the characteristic peaks of C_c and $C_{c'}$ on O-CH_2 in five-membered and six-membered acetal rings.

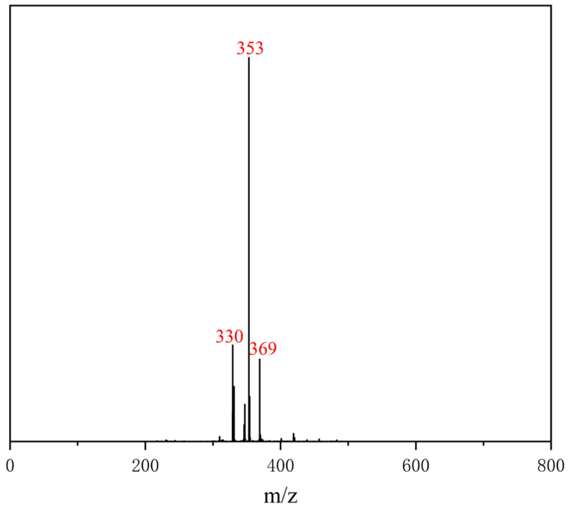

Figure S3. MALDI-TOF-MS spectrum of p-BBP.

As shown in Figure S3, the mass spectrum of p-BBP shows that the theoretical molecular weight is 330 g mol^{-1}, the measured $[M]^+$ is 330 g mol^{-1}. The difference between 353 g mol^{-1} and the theoretical value is 23 g mol^{-1}, which is $[M+Na^+]$. The difference between 369 g mol^{-1} and the theoretical value is 39 g mol^{-1}, which is $[M+K^+]$.

The molecular formula of p-BBP is $C_{18}H_{18}O_6$ and its molecular weight is 330.11. The theoretical contents of C and H are 65.49% and 5.50%, while the actual contents are 65.28% and 5.68%, and the deviation is within 0.3%, which shows that the synthesized p-BBP meets the expectations.

Curing behavior of m-p-BBP-a, m-an-p-BBP-a and m-ph-p-BBP-a

Figure S4 and Figure S5 are the segmented curing infrared curves of m-p-BBP-a and m-p-ph-BBP-a respectively. It can be seen that with the increase of temperature, the changes of m-p-BBP-a and m-an-p-BBP-a are the same, however, the terminal oxazine ring of m-ph-p-BBP-a was opened, which was consistent with the higher curing temperature. The curing mechanism is shown in Scheme S1.

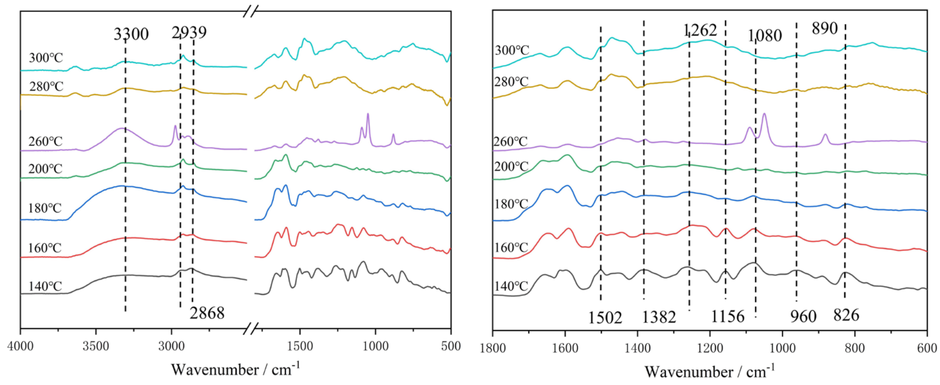

(a) (b)

Figure S4. FT-IR spectrum of m-p-BBP-a at different temperatures: (a) wavenumber range from 4000 to 500 cm^{-1}; (b) wavenumber range from 1800 to 600 cm^{-1}.

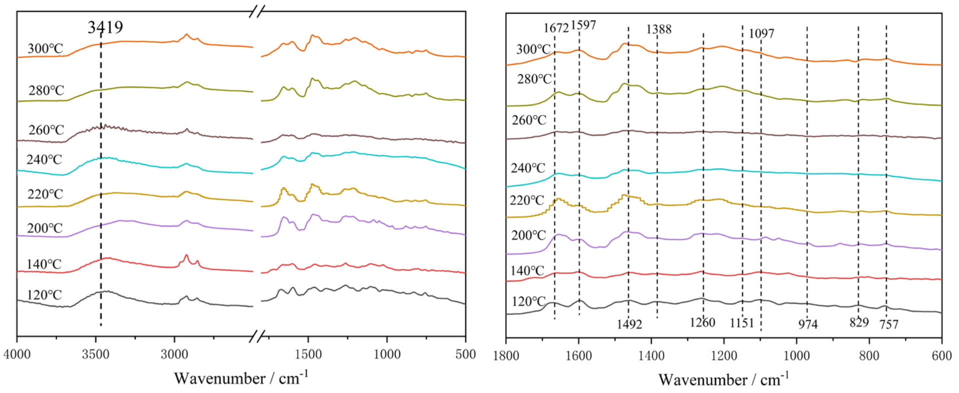

(a) (b)

Figure S5. FT-IR spectrum of m-ph-p-BBP-a at different temperatures: (a) wavenumber range from 4000 to 500 cm^{-1}; (b) wavenumber range from 1800 to 600 cm^{-1}.

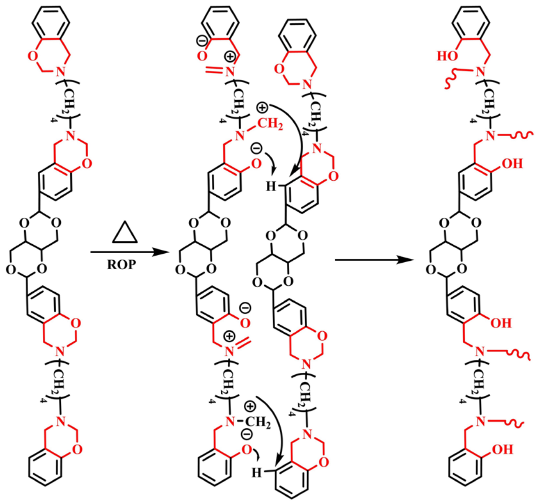

Scheme S1. Thermal ring-opening curing mechanism of m-ph-p-BBP-a.

Based on the analysis of curing kinetics of three benzoxazine-based resins, Table S1 shows the DSC parameters of non-isothermal curing of benzoxazine-based resins with different heating rates, and Figure S6 shows the liner fit of benzoxazine-based resins curing temperature.

β is the heating rate, T_i is the initial curing temperature, T_P is the peak curing temperature, T_f is the termination curing temperature, and ΔT is the curing interval; -ΔH is the enthalpy of curing.

Table S1. The non-isothermal curing DSC data of benzoxazine-based resins.

BZ	β /k/min	T_i/k	T_p/k	T_f/k	ΔT/k	-ΔH/J/g
m-*p*-BBP-a	5	373.71	461.95	546.92	173.21	315.2
	10	372.02	470.72	547.60	175.58	313.9
	15	375.06	472.75	558.06	183.00	309.3
	20	374.72	475.58	566.50	191.78	304.3
m-an-*p*-BBP-a	5	393.63	457.91	561.10	167.47	217.6
	10	395.66	467.49	559.41	163.75	210.3
	15	401.74	472.06	568.53	166.79	209.3
	20	401.06	476.54	564.48	163.42	207.9
m-ph-*p*-BBP-a	5	368.31	480.17	539.15	170.84	163.9
	10	372.70	487.26	549.96	177.26	178.7
	15	371.35	486.13	558.40	187.05	193.2
	20	378.10	493.18	566.17	188.07	183.6
	25	384.95	485.99	573.15	188.20	187.5

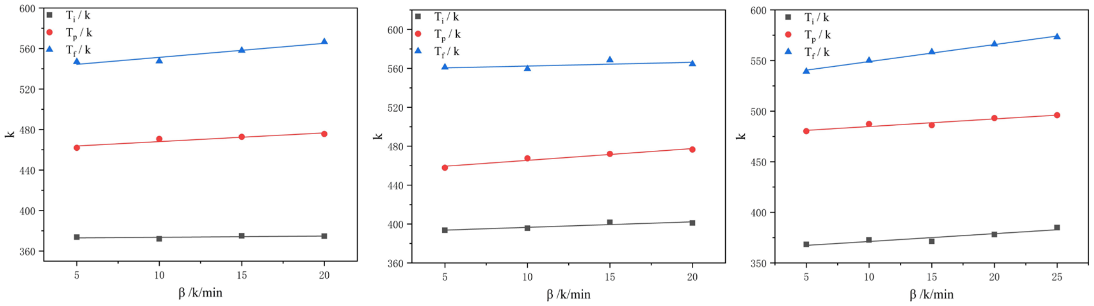

(a) (b) (c)

Figure S6. Liner fit of benzoxazine-based resins curing temperature: (a) m-p-BBP-a; (b) m-an-p-BBP-a; (c) m-ph-p-BBP-a.

Curing temperature is obtained from curve intercept, as shown in Table S2.

Table S2. The curing temperature of benzoxazine-based resins.

BZ	T_i/k	T_p/k	T_f/k
m-*p*-BBP-a	372.36	459.52	537.47
m-an-*p*-BBP-a	390.93	453.39	558.57
m-ph-*p*-BBP-a	363.48	477.28	532.10

The curing process of three benzoxazines was analyzed by model fitting method. The curing parameters of non-isothermal DSC are shown in Table 6.

Table S3. The non-isothermal DSC curing parameters of benzoxazine-based resins.

BZ	β/k/min	T_P/k	1000/T_P	-ln/β/T_P^2	lnβ
m-*p*-BBP-a	5	461.95	2.164736	10.66148	1.609438
	10	470.72	2.124405	10.00594	2.302585
	15	472.75	2.115283	9.609083	2.70805
	20	475.58	2.102696	9.333338	2.995732
m-an-*p*-BBP-a	5	457.91	2.183835	10.64391	1.609438
	10	467.49	2.139083	9.992171	2.302585
	15	472.06	2.118375	9.606162	2.70805
	20	476.54	2.09846	9.337371	2.995732
m-ph-*p*-BBP-a	5	480.17	2.082596	10.73884	1.609438
	10	487.26	2.052292	10.07501	2.302585
	15	486.13	2.057063	9.664902	2.70805
	20	493.18	2.027657	9.406016	2.995732
	25	495.99	2.016170	9.194236	3.218876

The related calculation results of benzoxazine are shown in Table 4-8.

Table S4. result computation.

BZ	β /k/min	S	n	A
m-*p*-BBP-a	5	1.61	1.60	3.16E+19
	10	1.60	1.59	2.60E+19
	15	1.80	1.69	3.20E+19
	20	1.95	1.76	3.23E+19
	average value	1.74	1.66	3.05E+19
m-an-*p*-BBP-a	5	1.10	1.32	2.06E+14
	10	0.82	1.14	1.97E+14
	15	1.18	1.37	2.10E+14
	20	1.12	1.33	2.02E+14
	average value	1.29	1.29	2.04E+14
m-ph-*p*-BBP-a	5	0.59	0.97	7.01E+20
	10	0.64	1.01	6.71E+20
	15	0.91	1.20	1.13E+21
	20	0.73	1.08	7.36E+20
	25	0.96	1.24	3.33E+19
	average value	0.77	1. 10	3.59E+19

Rheological analysis of benzoxazine-based resins

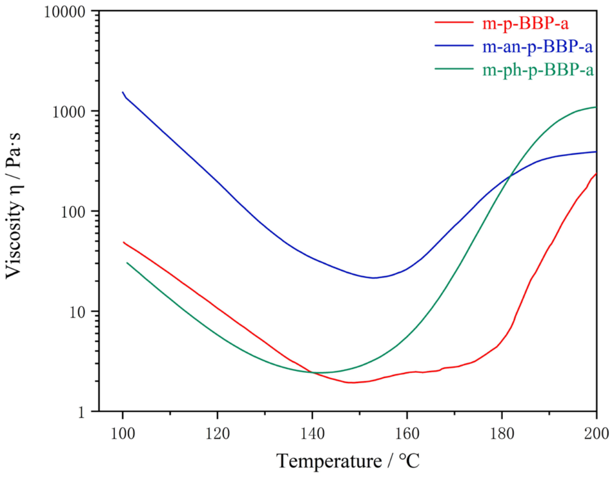

Figure S7. Relationship between viscosity and temperature of benzoxazine-based resins (m-p-BBP-a, m-an-p-BBP-a, and m-ph-p-BBP-a).

The relationship between viscosity and temperature of benzoxazine-based resins is shown in Figure S7. It can be seen that all benzoxazine-based resins show the same development trend, which can be roughly divided into two stages. In the first stage, the temperature increases and the viscosity decreases, when the benzoxazine-based resins are significantly affected by the temperature, the temperature plays a leading role, and the chemical active groups are slow to react, then the viscosity is determined by the temperature. The temperature continues to rise, which is beneficial to chemical crosslinking reactions and can increase viscosity. When it reaches a balance with the viscosity reduction caused by temperature, it enters the second stage. With the increase in temperature, the influence of chemical crosslinking reaction on viscosity gradually exceeds the temperature, and at this time, the viscosity is determined by chemical reaction, which leads to the rapid increase of viscosity. The viscosity of m-ph-p-BBP-a began to rise around 140°C, and it began to rise rapidly around 170°C, and the viscosity increase was the most obvious. The reason may be that the phenolic hydroxyl group in the molecule is beneficial to its curing and crosslinking. M-ph-p-BBP-a has a very low viscosity at low temperatures, but it can rise rapidly when it is raised to a certain temperature, which shows that the resin has better processability.

Reference:

1. Yuan, W.; Ma, S.; Wang, S.; Li, Q.; Wang, B.; Xu, X.; Huang, K.; Chen, J.; You, S.; Zhu, J. Synthesis of fully bio-based diepoxy monomer with dicyclo diacetal for high-performance, readily degradable thermosets. *Eur Polym J* **2019**, 117, 200-207. [CrossRef]
